# Primary Versus Iatrogenic Spondylolisthesis: A Multi-Dimensional Comparison of Outcomes

**DOI:** 10.3390/jcm14072193

**Published:** 2025-03-23

**Authors:** Dana-Georgiana Nedelea, Diana Elena Vulpe, Serban Dragosloveanu, Ioan Cristian Stoica

**Affiliations:** 1 Doctoral School, The “Carol Davila” University of Medicine and Pharmacy, 050474 Bucharest, Romania; dana-georgiana.nedelea@drd.umfcd.ro; 2 Department of Orthopaedics, “Foisor” Clinical Hospital of Orthopaedics, Traumatology and Osteoarticular TB, 021382 Bucharest, Romania; cristian.stoica@umfcd.ro; 3 Department of Orthopaedics and Traumatology, The “Carol Davila” University of Medicine and Pharmacy, 050474 Bucharest, Romania

**Keywords:** spondylolisthesis, orthopedic surgery, spine surgery, low back pain, leg pain, disability, rehabilitation, imaging

## Abstract

**Background**: Spondylolisthesis is an important cause of lumbar and/or leg pain and can occur primarily or following spinal surgery. Our study aimed to compare the outcomes, patient satisfaction, and complications in patients surgically treated for primary versus iatrogenic spondylolisthesis. **Methods**: We included 90 patients who underwent spinal surgery for primary (group A, n = 46) and iatrogenic (group B, n = 44) spondylolisthesis. Radiographs were performed before and after spinal surgery. Low back pain and leg pain were assessed with the Visual Analog Scale preoperatively, postoperatively, and at 12 months, the Frankel classification was used to assess neurological impairment, and the Oswestry Disability Index was assessed preoperatively at 6 and 12 months. **Results**: Patients in group A had better surgical outcomes, with shorter surgical time (*p* = 0.005), less intraoperative bleeding (*p* = 0.0015), and achieving higher degrees of spondylolisthesis reduction (*p* = 0.0128) with more reduction distance reached (*p* = 0.0153). Moreover, patients from group A had significantly higher levels of low back pain preoperatively compared to patients from group B (*p* = 0.0042). No significant differences were noted in low back pain and leg pain at the 6- and 12-month follow-ups. Fewer implant failures were seen in group B, while group A had a slightly faster arthrodesis rate. **Conclusions**: Iatrogenic spondylolisthesis requires additional surgeries with increased risks and socioeconomic costs. However, while surgical challenges differ based on the etiology of spondylolisthesis, the long-term outcomes might not vary significantly. Future research is needed to address the optimization of surgical techniques and rehabilitation strategies in order to improve the outcomes in both cohorts.

## 1. Introduction

Spondylolisthesis, or the slippage of one vertebra over the other, can be diagnosed at any age with a variety of underlying causes and represents one of the most important reasons for seeking medical care. Several classifications have attempted to best describe this pathology, mainly based on the cause or the degree of the slippage. This complex pathology is frequently associated with chronic symptoms such as low back pain and/or leg pain [1]. The most common type of spondylolisthesis is isthmic, usually at the L5-S1 level, followed by degenerative and dysplastic [2]. The presentation of spondylolisthesis can vary widely from asymptomatic to different degrees of back and leg pain to extremely severe cases with cauda equina syndrome [3].

Iatrogenic spondylolisthesis is a well-known complication of different spinal interventions, especially when the normal spinal biomechanics are altered. It was first described in 1976 by Wiltse and White [4], and was defined as the translational shifting on radiographic examination of one vertebral segment over an adjacent one at the same level where a previous spinal procedure was performed [5].

While simple laminectomy is the most common procedure for disc hernia and symptomatic spinal stenosis, performed to decrease pain and reinstate function, the normal spinal biomechanics may be altered, predisposing to iatrogenic instability and spondylolisthesis [6]. Post-laminectomy segmental spinal instability is one of the most prevalent indications for reoperation [7,8]. Essential components of the posterior ligamentous complex need to be protected in order to maintain normal spinal stability, as well as preoperative assessment of the facet joints and disc morphology in cases anticipated to have iatrogenic instability [9,10]. The use of newer, minimally invasive techniques such as the full-endoscopic laminotomy provides better outcomes regarding the incidence of iatrogenic spondylolisthesis compared with the conventional subtotal laminectomy [11].

Iatrogenic instability significantly affects clinical outcomes, with a high risk for revision surgery, which is associated with higher complication rates, lower satisfaction rates, and increased medical costs [12]. Comparative literature data on primary and iatrogenic spondylolisthesis and their outcomes are scarce. A recent study showed that that outcomes of primary lumbar fusions are better than those from revisions [13], however, additional information is needed regarding the impact of various demographic, clinical, intraoperative, and perioperative factors on the outcome of these patients. This would allow for a comprehensive view, which could lead to improved and, perhaps, personalized management of primary versus iatrogenic spondylolisthesis [14].

In this study, we aimed to assess the difference between primary and iatrogenic spondylolisthesis in terms of surgical parameters, the improvement in low back pain and leg pain after surgery, patient overall satisfaction, and complications experienced in patients surgically treated for spinal segmental instability.

## 2. Materials and Methods

### 2.1. Study Design

Our retrospective study included 90 patients admitted for the treatment of spondylolisthesis between November 2016 and October 2023 in the “Foisor” Clinical Hospital of Orthopedics, Traumatology, and Osteoarticular TB. All patients were submitted to surgery. Two groups were formed. The first group (group A) included consecutive patients admitted with primary spondylolisthesis, and the second group (group B) included patients admitted with iatrogenic spondylolisthesis.

The inclusion criteria for group A were the following: patients with back pain or/and leg pain associated with imaging findings of spondylolisthesis who had already undergone 3 months of conservative treatment without satisfactory results, and patients with back pain or/and leg pain associated with imaging findings of spondylolisthesis, and neurological impairment. Conservative treatment consisted of lifestyle change, 2 weeks of anti-inflammatory and pain medication, and inclusion in a three-month physical therapy and rehabilitation program. Back pain and leg pain were assessed with the Visual Analog Scale (VAS) at the beginning of conservative treatment and compared with scores obtained at the end of the 3 months. If a difference of less than 3 points was noted between the two scores, patients were submitted for surgical treatment and included in group A. Therefore, group A consisted of consecutive patients admitted with a diagnosis of primary spondylolisthesis and surgical indication.

Inclusion criteria in group B consisted of patients presenting to the ambulatory care unit or were referred to our hospital by their primary care physician or another hospital for back pain or/and leg pain associated with imaging findings of spondylolisthesis and history of previous spinal decompression who had already undergone 3 months of conservative treatment without satisfactory results and patients with back pain and/or leg pain, with imaging findings of spondylolisthesis, history of previous spinal interventions, and neurological impairment. The same 3 months conservative treatment protocol was used for the patients in both groups, with the same patient satisfaction assessment.

All patients signed an informed consent confirming their agreement to participate in the study. The research was conducted under the ethical principles for medical research in accordance with the Declaration of Helsinki from 1964 and its later amendments. This study was approved by the “Foisor” Clinical Hospital of Orthopedics, Traumatology, and Osteoarticular TB Ethical Council with registration number 924/31 January 2025.

### 2.2. Evaluation After Hospital Admission

After admission to the hospital, the patients were evaluated by the same team of spinal specialists. General data collected on the patients included gender, age, body mass index (BMI), smoking habits, location of spondylolisthesis, descriptive classification of spondylolisthesis according to Wiltse et al. [15] and severity of the slippage classification according to Meyerding [16]. The spinal specialist evaluated the low back pain and leg pain of every patient preoperatively using the Visual Analog Scale (VAS) [17] and the neurological impairment was assessed using the Frankel classification [18]. The Oswestry Disability Index (ODI) was also assessed preoperatively [19].

All patients were submitted to surgery and the following data were assessed: the total surgical time (meaning the time after the anesthesia induction until the last thread was positioned), intraoperative bleeding, hospital stay length, number of fused levels, achieved reduction in spondylolisthesis, and grade reduction according to Meyerding’s classification [16]. The postoperative low back pain and leg pain were assessed using the VAS, postoperative neurological impairment was evaluated using the Frankel classification, and the postoperative ODI was also applied. Follow-up included clinical and radiological examinations at 6 and 12 months after surgery, and the achieved arthrodesis was assessed in number of months after surgery and quantified as present or absent on repeated follow-up radiographic examinations performed by the same team of spinal specialists. The implant failure, whether it involved a rod or screws, was also evaluated, along with the low back pain, the leg pain, and the ODI.

At admission, blood samples were drawn for all patients to prepare them for surgery. All patients benefited from an X-ray examination and Magnetic Resonance Imaging (MRI) of the spine. For cases in which an MRI could not be obtained a CT was performed.

### 2.3. Image Acquisition and Analysis

All patients underwent anteroposterior (AP) and lateral (LL) spine X-rays, along with the specialized projections that provide functional tests of spinal instability in our Department of Radiology and Medical Imaging. The examinations were performed on a DigitalDiagnost R3.1 machine (Philips Medical Systems Nederland B.V., Amsterdam, The Netherlands). Examinations were performed with the patient erect and in suspended expiration. For the anteroposterior projection, patients were examined with arms along the sides using an exposure protocol of 70–80 kVp and 80–110 mAs. For the lateral view, the patient was examined with arms extended to the front and an exposure regimen of 70–90 kVp and 60–100 mAs.

The specialized projections used as functional tests of lumbar spine instability were the lumbar spine flexion and extension lateral views, which were done after the classical lateral view of the lumbar spine and consisted of instructing the patient to “bend forward” as much as they could for the flexion lateral view, or “lean back” for the extension lateral view. The exposure parameters for these specialized projections were similar to the ones for the lateral view of the lumbar spine. After surgery, the anteroposterior (AP) and lateral (LL) spine radiographs were repeated immediately and at intervals of 3, 6, and 12 months according to hospital protocol. All views were performed with an anti-diffusion X-ray grid and a focus-film distance of 115 cm. The digital images were verified for quality and stored in the hospital’s archiving and communication system (PACS). All images were accessed through the PACS, viewed using a dedicated radiology monitor, and analyzed using MediCAD 7.0.4.4543 (Hectec GmbH, Altdorf, Germany).

**Figure 1:**
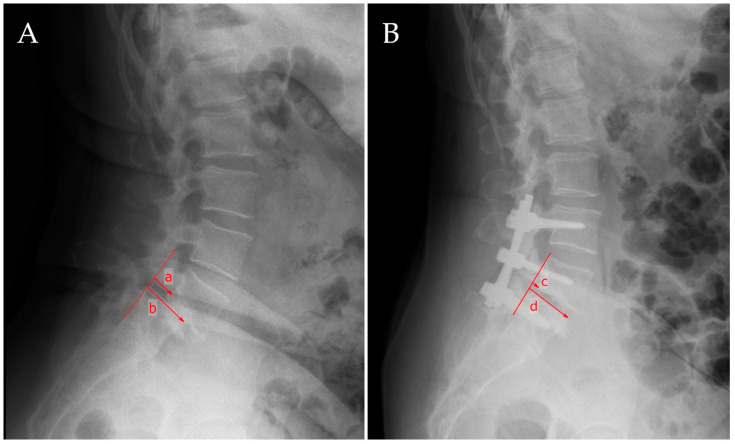
Measurement of pre-and postoperative spondylolisthesis slippage. A vertical line was drawn through the posterior margin of the caudal vertebra. On preoperative assessment (**A**), a is the distance from this vertical line to the posterior margin of the upper vertebra, while b is the length of the cranial endplate of the caudal vertebra, c is the distance from the line drawn vertically through the posterior margin of the vertebra below to the posterior wall of the upper vertebra, and d is the length of the superior endplate of the below vertebra assessed postoperatively (**B**). The preoperative slippage is defined as a/b, whereas the postoperative slippage is defined as c/d.

For the assessment of spondylolisthesis, we performed several measurements as depicted in Figure 1: On the lateral projection, a line was drawn alongside the posterior wall of the two adjacent vertebral bodies that constituted the slippage, allowing for the evaluation of vertebral translation, and a measurement in centimeters of the actual slippage was performed. Measurements were assessed pre- and postoperatively.

**Figure 2:**
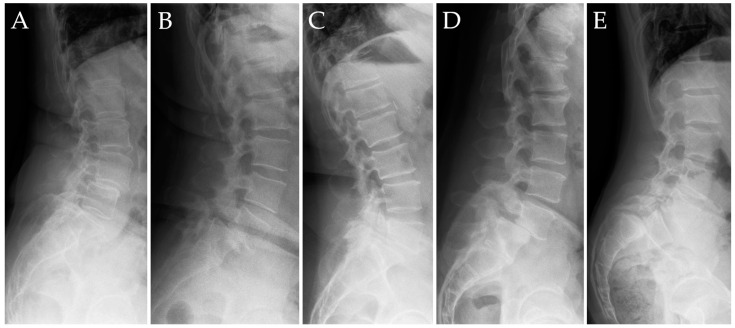
Meyerding’s classification was exemplified in 5 patients from our cohorts. (**A**) grade I: 0–25% slippage. (**B**) grade II: 25–50% slippage. (**C**) grade III: 50–75% slippage. (**D**) grade IV: 75–100%. (**E**) grade V: greater than 100% slippage [16].

We used the Meyerding classification of the degree of vertebral slippage to analyze the preoperative spondylolisthesis grade and the postoperative result, with referral to the achieved correction in grade and millimeters [16] (Figure 2).

All patients in this study underwent surgical treatment consisting of Posterior Lumbar Interbody Fusion (PLIF) and were operated on by the same spinal surgical team. Patients were in a prone position on the operating table. A midline approach centered on the affected region of the spine was performed, with a bilateral muscle strip dissection in order to gain access to the posterior column of the vertebral body. A Pedicle screw fixation was performed under fluoroscopy at the preoperative planned levels. A laminectomy at the appropriate level was then performed, the dura was retracted, and access to the intervertebral disc space was acquired. The disc space and the endplates were prepared to allow for intervertebral implant insertion. The interbody cage insertion was performed under fluoroscopic control. Then, rods were implanted, and a final verification of the instrumentation was performed.

On the first day after surgery, every patient was consulted by a rehabilitation and physical therapy specialist and included in a rehabilitation and physical therapy program. This program included correct mobilization and walking without assistance for the first two weeks, then progressively introducing easy exercises until 6 weeks after surgery. After the radiographic follow-up at 6 weeks, physical therapy for toning the lumbar paravertebral muscles was started.

Follow-ups at 6 and 12 months after surgery were scheduled, and the VAS for back and leg pain, and the ODI Index were noted. There was no recorded loss to follow-up.

### 2.4. Statistical Analysis

The patient data were recorded in table format and analyzed using MedCalc^®^ Version 14.8.1 (MedCalc Software bvba, Ostend, Belgium). The values were tested for normality using the Shapiro–Wilk test for continuous variables. The differences between groups for continuous variables with normal distribution were tested with a Student t-test, and for dichotomic variables were assessed with Fisher’s exact test. *p* values were considered statistically significant when values were lower than 0.05. Values were reported as mean value ± standard deviation.

## 3. Results

The study included a total of 90 patients, with group A (primary spondylolisthesis) comprising 46 patients and group B (iatrogenic spondylolisthesis) comprising 44 patients. Table 1 provides an overview of the demographic characteristics of the studied population.

A significant difference was noted in the age of the patients, with group A consisting of younger patients (mean age of 59.80 ± 12.36 years old) than group B (mean age of 64.95 ± 10.02 years old). The location of the spondylolisthesis was noted, and most patients had either L4-L5 or L5-S1 levels affected. According to Witse’s classification, group A consisted of 8 isthmic spondylolisthesis and 38 degenerative cases, and group B consisted of 44 iatrogenic cases. Regarding the severity of the slippage, according to Meyerding’s classification, most cases were type II in both groups, some were type III, and only 2 cases were type IV (Table 2).

Parameters registered in the operating room consisted of the surgical time (in minutes), counted from the anesthesia induction until the last thread (in minutes), and intraoperative bleeding (in milliliters). All patients were operated on by the same surgical team. Significant differences were noted in these two parameters, with group A registering less surgical time (*p* = 0.0005) and less intraoperative bleeding (*p* = 0.0015) than group B, but with no significant difference regarding the hospital length of stay (in days) between groups. Patients in group A had better surgical outcomes, achieving higher degrees of spondylolisthesis reduction (*p* = 0.0128) and more reduction distance reached (*p* = 0.0153), as indicated in Table 3.

The VAS was used to evaluate low back pain and leg pain both preoperatively and postoperatively, and it was performed by the same spinal specialist to ensure reliability and minimize bias. After a thorough analysis of the acquired data, patients from group A had significantly higher levels of low back pain preoperatively (*p* = 0.0042) than patients from group B (Table 4).

The ODI was evaluated before surgery and at the 6- and 12-month follow-ups, with similar results recorded between the two groups. Furthermore, no significant differences were noted in low back pain and leg pain at the 6- and 12-month follow-ups (Table 5). While statistically significant differences were noted in all the surgery-related data, such as the surgical time and the intraoperative bleeding, and better reduction both in millimeters and grade were achieved in the primary group, no clinically meaningful implications were evident, as no differences in outcomes and patient satisfaction were recorded at the 6- and 12-month follow-ups.

**Figure 3:**
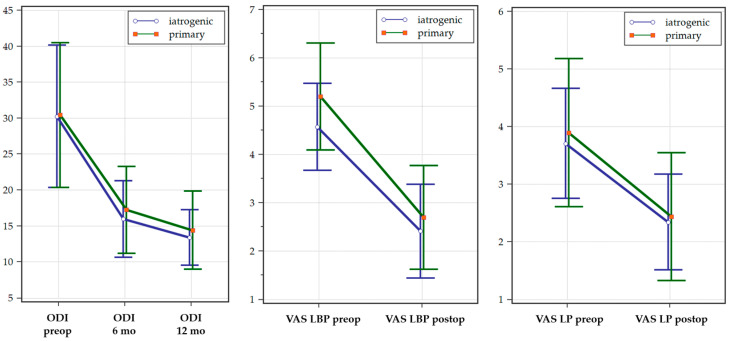
Evolution of the Oswestry Disability Index (ODI), low back pain (LBP) and leg pain (LP), between the two study groups at different time points.

A graphical representation of the evolution of pain and disability scores between the two lots at the different time points assessed is provided in Figure 3.

In terms of implant failure, we have encountered four screws and one rod failure in group A and one screw and one rod failure in group B. Although more implant failures were registered in the primary spondylolisthesis group, no significant difference was noted between the two (*p* = 0.3611 for screw failure, and *p* = 1.0000 for rod failure) (Table 6).

The presence of arthrodesis and the time interval from the surgery (in months) were assessed on radiographic imaging by the same spine specialist. There were no statistically significant differences in the time interval between the surgery and the spinal arthrodesis encountered, although patients in group A achieved faster arthrodesis than patients in group B (11.98 ± 1.16 vs. 13.09 ± 4.12 months, *p* = 0.0912). For patients in group B, the time interval from the surgery between primary and iatrogenic spondylolisthesis varied between 6 and 120 months and averaged 37.20 ± 23.28 months.

## 4. Discussion

One of the most frequently performed spinal interventions is represented by laminectomy with good results and sustained long-term outcomes. However, the incidence of iatrogenic spondylolisthesis is cited in the literature as being between 0 and 63% [20,21]. A new literature review that comprised 1257 patients suggested that 1.6% to 32% of patients who underwent open lumbar decompression developed new or aggravated spondylolisthesis that required fusion [22]. Postoperative mechanical spinal instability and spondylolisthesis after decompression are some of the most frequent indications for reoperation and spinal arthrodesis, but this procedure carries increased surgical risks [7]. Numerous studies highlight the role of the posterior osteo-ligamentous structures and the link between bilateral laminectomy and enlarged segmental mobility [23,24]. In a systematic review of 2496 patients treated with open laminectomy and newer minimally invasive techniques, postoperative instability was noticed in patients who had spondylolisthesis, as well as those undergoing open laminectomy [25].

After a thorough analysis of all 44 patients from the iatrogenic spondylolisthesis group, paired with a group of patients diagnosed with primary spondylolisthesis, results showed an increased surgical risk, with higher values for surgical time intervention and higher blood loss in the first group of patients. These differences were probably caused by the fact that this was a second intervention at the same level, with fibrous tissue scar attached to the posterior elements and the dura, thus making the approach and all interventions at the affected level more difficult [26].

Increased surgical time was also encountered in other studies on iatrogenic spondylolisthesis. König et el. reported in their case series on iatrogenic spondylolisthesis patients that the mean operative time, including anesthesia, was 223 ± 26min (range between 200 and 300 min). In the same paper, blood loss was also assessed, with a mean blood loss of 284 ± 76.6 mL (range between 200 and 400 mL) [27].

Increased surgical time and blood loss were associated with increased risks of complications, such as infection and increased non-union rates, in almost every paper regarding spinal surgeries. In a recent study of 336 patients, increased operative time was significantly associated with a higher rate of complications. Moreover, for surgical times of less than 120 min, the complication rate was calculated at 2.4%, whereas for 120 to 240 min, the complication rate was 44.5%. Surgery lasting 240 to 360 min had a complication rate of 69.5%, whereas prolonged surgical time of more than 360 min had a complication rate of 85.7% [28].

In a study on 112 patients with revision posterior lumbar spine decompression, fusion, and segmental instrumentation, intraoperative blood loss and operative time were assessed. After analyzing the results of the multiple regression analysis, it seems that the most significant factors predicting these were the number of levels fused and age [29].

In our study, better reduction, both in millimeters and Meyerding’s grading, was achieved in the primary spondylolisthesis group. It may prove extremely difficult to release scarring tissue from previous spinal surgery, thus obtaining less reduction in iatrogenic spondylolisthesis. We sought to obtain an equally large patient specimen in both primary and iatrogenic spondylolisthesis. The current literature on the surgical treatment of iatrogenic spondylolisthesis is less consistent, and a comparison between complications of iatrogenic spondylolisthesis versus primary spondylolisthesis has rarely been analyzed. To our knowledge, no prior studies have researched this issue. In spite of a better reduction in spondylolisthesis achieved in the primary group, no significant differences were noted between the two groups for the mid-term follow-up in terms of patient satisfaction and outcomes, as demonstrated by the scores achieved at the 6- and 12-month follow-ups in both groups.

Patients who were operated on for iatrogenic spondylolisthesis were older than those who received surgical treatment for primary spondylolisthesis because there was a time interval between the first spinal intervention of laminectomy and the second one, including arthrodesis. The time interval between the decompression surgery and the arthrodesis of the spine for our cohorts was established between 6 and 120 months, with the majority of patients needing the second surgery between one to three years after decompression. In another case series of 105 patients with laminectomies, 10 patients developed iatrogenic spondylolisthesis, and the mean period between the first and second surgical intervention was noted to be 32.6 ± 19.9 months [22].

BMI was not associated with differences between groups. Patients in group A had a mean BMI of 24.91 ± 2.67, and patients in group B had a mean BMI of 29.87 ± 2.61. In a study that analyzed the sagittal spinopelvic alignment and the body mass index (BMI) in a group of patients with spondylolisthesis, compared to a group of people with low back pain without spondylolisthesis, results showed that the average BMI was significantly higher (*p* = 0.03) in the spondylolisthesis group, with 71.4% of the spondylolisthesis patients being overweight, with a BMI of more than 25 kg/m^2^ [30]. Multiple other studies have researched the role of BMI on spondylolisthesis, with contradictory results. Jacobsen et al. published their results in which higher BMI was associated with more frequent slips, but concerning only women [31]. The presence of a relationship between BMI and spondylolisthesis is noted by many authors in distinct populations and different sample sizes [32,33,34].

A significant difference was noted in the VAS related to back pain, as the patients who received surgical treatment for primary spondylolisthesis scored higher levels of pain before surgery compared to the patients from the iatrogenic group. This may have been associated with higher pain tolerance in the iatrogenic group, as their disease was progressing over a long period. No noted difference between VAS scores for leg pain before surgery, back pain, or leg pain after surgery between the two groups.

In the long term, no difference was noted in back or leg pain 12 months after surgery between groups. The ODI showed similar patient satisfaction between groups at the 6- and 12-month follow-ups. Similar results were also noted in the literature for long-term follow-up [35,36,37].

Implant failure was noted between the two groups, and more cases presented this complication in the primary group than in the iatrogenic group. Most implant failures included screw failure, probably due to a combination of mechanical stress and poor bone quality. In a study on 40 patients operated on for high-grade spondylolisthesis, 17% of symptomatic non-union was found to be associated with deterioration in the slippage, screw breakage, and graft fracture [38]. Molinari et al. compared three different surgical techniques for high-grade spondylolisthesis and found that the incidence of pseudarthrosis was 45% in the group that had an L4-S1 posterior fusion without decompression, 29% in the group that had decompression with posterior instrumentation and fusion, and 0% in the group with circumferential fusion procedure [39].

The limitations of our study include the small number of patients included, as well as its retrospective design, as this limits our demographic data to the parameters already registered in the hospital archive. Another limitation is the lack of imaging findings from the first surgery for the patients in the iatrogenic spondylolisthesis group, as they reported to our hospital with symptomatic mechanical instability after being operated on in a different facility. As preoperative spondylolisthesis is an important predictor of iatrogenic instability, the pre-existing spinal instability should have been assessed before the first surgery [10,40]. Identifying spondylolisthesis prior to surgical decompression of the spine is associated with a higher incidence of iatrogenic spondylolisthesis and one of the most frequent reoperation indications, with associated important risks, and socioeconomic costs [37]. A longer follow-up period would be beneficial for better-assessing results, and using other measurements for functional outcomes would provide a better perception of the actual benefit of surgical treatment.

## 5. Conclusions

Our study revealed key differences in the demographic, surgical, and clinical factors between patients with primary and iatrogenic spondylolisthesis. Iatrogenic spondylolisthesis following spinal surgeries is an important complication that requires additional surgeries with increased risks and socioeconomic costs. In patients with primary spondylolisthesis, a decompression-only approach will lead to further instability due to disruption of the posterior osteo-ligamentous structures, especially in those with a high BMI. Patients with recurrent symptoms of radiculopathy at various amounts of time following a spinal decompression should be assessed for iatrogenic spondylolisthesis. Future research is needed to accurately assess the areas of improvement in surgical techniques, as well as to propose rehabilitation strategies that are required to improve clinical outcomes in patients with spondylolisthesis regardless of the etiology.

## Figures and Tables

**Table 1:** Demographic parameters in the studied population.

Parameter	Group A—Primary	Group B—Iatrogenic	*p* Value
Age	59.80 ± 12.36	64.95 ± 10.02	0.0334
Body Mass Index (BMI)	29.41 ± 2.67	29.87 ± 2.61	0.4147
Smoker status	30/46 (65.2%)	24/44 (54.5%)	0.3899

All values are presented as mean value ± standard deviation (SD).

**Table 2:** Anatomical variations of the affected levels of the lumbar spine, types of spondylolisthesis, and degree of vertebral slippage.

Parameter	Group A—Primary	Group B—Iatrogenic	*p* Value
Location			
L3-L4	3	1	0.3426
L4-L5	22	27	
L5-S1	21	16	
Type ^1^			
II	8	0	<0.0001
III	38	0	
V	0	44	
Degree of slippage ^2^			
II	29	35	0.1340
III	15	9	
IV	2	0	

^1^ according to Witse’s classification; ^2^ according to Meyerding’s classification.

**Table 3:** Intraoperative parameters registered and postoperative outcomes for both groups of the studied population.

Parameter	Group A—Primary	Group B—Iatrogenic	*p* Value
Surgical time (min)	151.44 ± 33.91	175.91 ± 29.75	0.0005
Intraoperative bleeding (mL)	568.00 ± 196.43	690.23 ± 152.25	0.0015
Spondylolisthesis after reduction ^1^			
• I	39	33	0.3701
• II	7	11	
Degree of reduction ^1^	1.26 ± 0.65	0.95 ± 0.48	0.0128
Distance of reduction (mm)	5.78 ± 1.88	4.93 ± 1.28	0.0153
Hospital length of stay (days)	6.00 ± 1.83	6.82 ± 2.96	0.1232

^1^ Meyerding’s classification of the degree of vertebral slippage; min = minutes; mm = millimeters.

**Table 4:** Low back pain and leg pain examination before and after surgery.

Parameter	Group A—Primary	Group B—Iatrogenic	*p* Value
Preoperative VAS			
Low back pain	5.20 ± 1.11	4.57 ± 0.90	0.0042
Leg pain	3.89 ± 1.19	3.70 ± 0.95	0.4378
Postoperative VAS			
Low back pain	2.70 ± 1.07	2.41 ± 0.97	0.1881
Leg pain	2.43 ± 1.11	2.34 ± 0.83	0.6521
Reduction in VAS			
Low back pain	2.50 ± 1.15	2.16 ± 0.89	0.1202
Leg pain	1.46 ± 1.52	1.36 ± 1.14	0.7444

VAS = Visual Analogue Scale.

**Table 5:** Follow-up at 6 and 12 months after surgery.

Parameter	Group A—Primary	Group B—Iatrogenic	*p* Value
ODI			
• preoperative	30.41 ± 10.06	30.23 ± 9.90	0.9299
• 6 months	17.24 ± 6.25	15.95 ± 5.30	0.2877
• 12 months	14.39 ± 5.43	13.38 ± 3.84	0.3117
VAS LBP 12 months	4.80 ± 1.22	4.52 ± 1.51	0.2640
VAS LP 12 months	4.13 ± 1.57	4.11 ± 1.48	0.9586

ODI = Oswestry Disability Index; VAS = Visual Analog Scale; LBP = low back pain; LP = leg pain.

**Table 6:** Implant failures in the study groups.

Parameter	Group A—Primary	Group B—Iatrogenic	*p* Value
Screw failure	4 (8.70%)	1 (2.27%)	0.3611
Rod failure	0	1 (2.27%)	1.000

## Data Availability

The data presented in this study are available on reasonable request from the corresponding author.

## Abbreviations

The following abbreviations are used in this manuscript:

AP	anteroposterior
BMI	body mass index
CT	Computed Tomography
LL	Latero-lateral
MRI	Magnetic Resonance Imaging
ODI	Oswestry Disability Index
PACS	Picture archiving and communication system
VAS	Visual Analog Scale
